# Supplementary material for: Boosting photocatalytic hydrogen production from water by photothermally induced biphase systems
Source: Nat Commun. 2021 Feb 26;12:1343. doi: 10.1038/s41467-021-21526-4 (PMC7910610; doi:10.1038/s41467-021-21526-4)
Supplement: Supplementary file 1 — Supplementary Information [file 41467_2021_21526_MOESM1_ESM.pdf]

Supplementary Information for

# Boosting Photocatalytic Hydrogen Production from Water by Photothermally Induced Biphase Systems

Shaohui Guo[1], Xuanhua Li[1]*, Ju Li[2], and Bingqing Wei[3]*

[1]State Key Laboratory of Solidification Processing, Center for Nano Energy Materials, School of Materials Science and Engineering, Northwestern Polytechnical University, Xi'an, 710072, China.

[2]Department of Nuclear Science and Engineering, Massachusetts Institute of Technology, Cambridge, MA, 02139, USA

[3]Department of Mechanical Engineering, University of Delaware, Newark, DE19716, USA

* Corresponding author E-mail:

Xuanhua Li lixh32@nwpu.edu.cn

Bingqing Wei weib@udel.edu

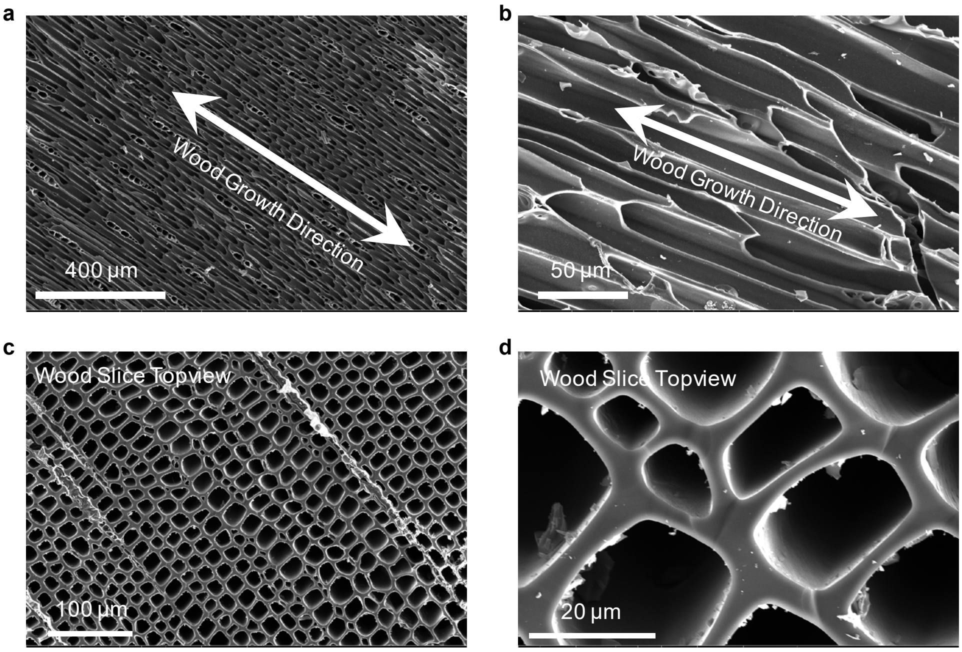


**Supplementary Figure 1.** SEM images of the wood slice. **a, b** Sideview SEM images of a pinewood. The pinewood growth direction has been observed. **c, d** Topview SEM images of the wood slice, which is cut from a tree perpendicular to its growth direction.

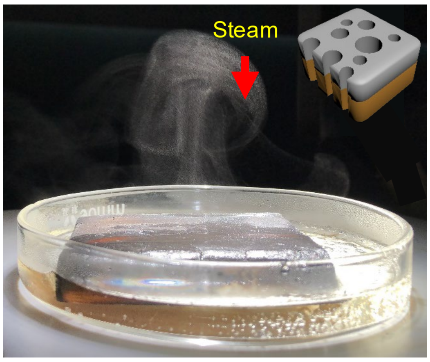


**Supplementary Figure 2.** Photograph of steam generation process from the surface carbonized wood under light illumination.

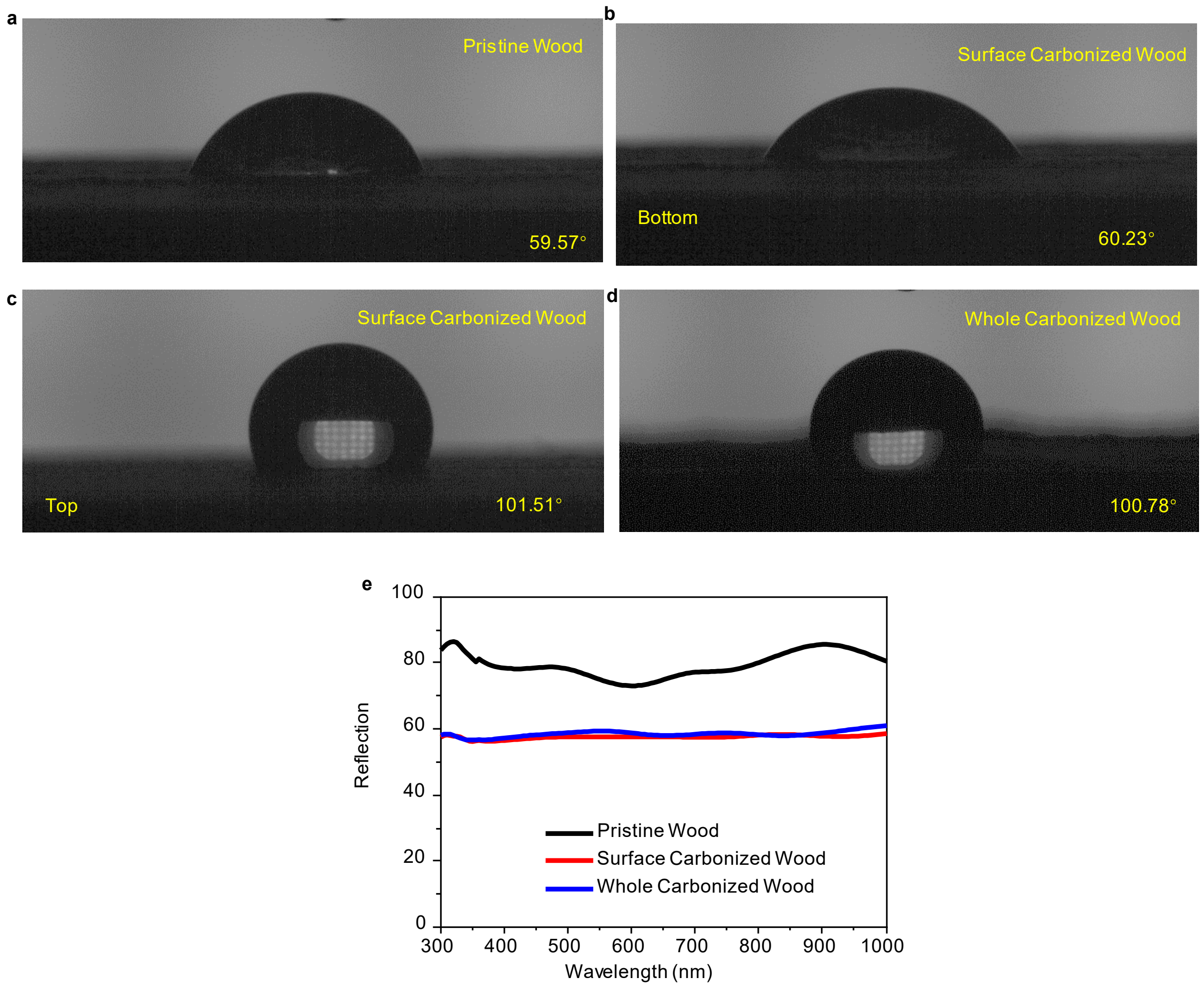


**Supplementary Figure 3. a-d** The contact angle measurement from the pristine wood, surface carbonized wood, and whole carbonized wood. **e** The reflection spectra of the pristine wood, surface carbonized wood, and whole carbonized wood.

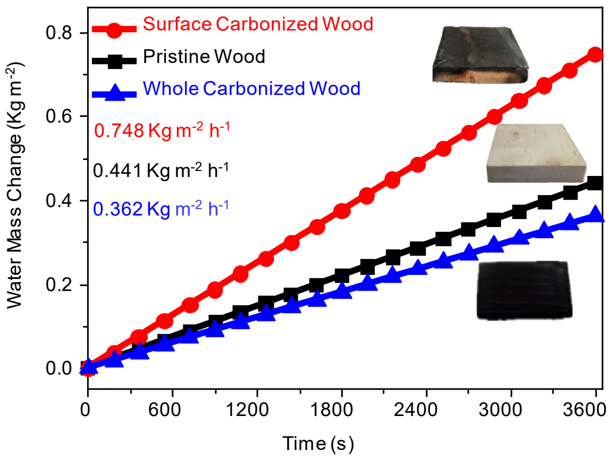


**Supplementary Figure 4.** Steam evaporation from wood induced mass changes in the water as a function of time. The inset image is the photograph of the pristine wood, surface carbonized wood, and whole carbonized wood. The surface carbonized wood shows the highest steam generation ability among three samples because of the good light absorptivity and the hydrophilic wood structure at the bottom.[1] The solar-to-steam conversion efficiencies of the pristine wood, surface carbonized wood, and whole carbonized wood are 27.65 %, 46.90 %, and 22.71 %, respectively.

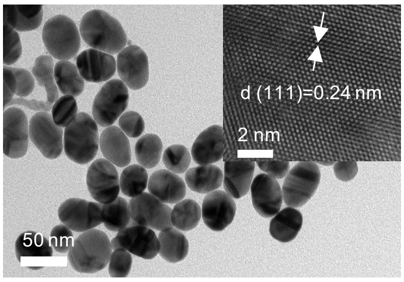


**Supplementary Figure 5.** TEM image of CoO NPs. Inset: HRTEM image of a CoO NP.

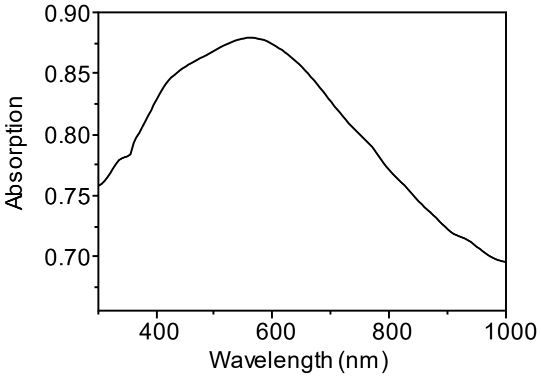


**Supplementary Figure 6.** The absorption spectra of CoO NPs.

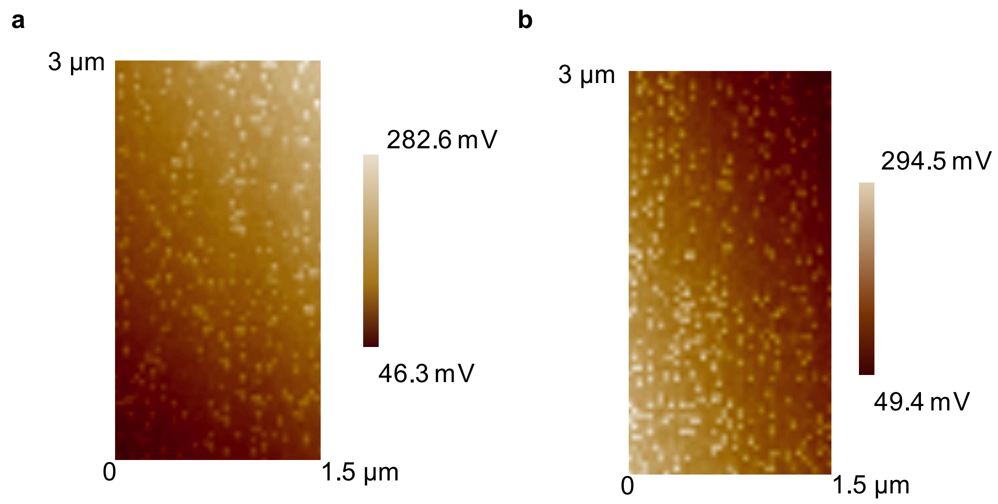


**Supplementary Figure 7.** **a** The potential of wood/CoO at 300 K, **b** the potential of wood/CoO at 308 K.

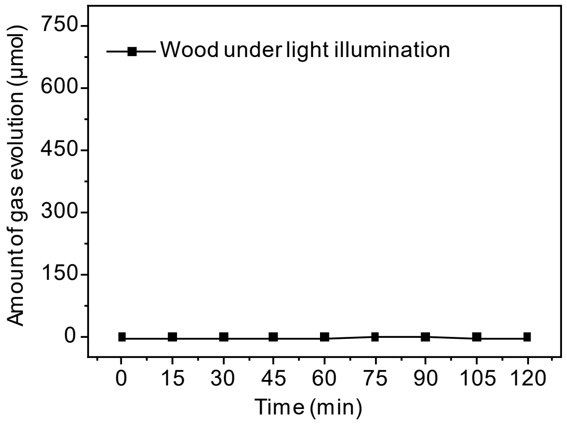


**Supplementary Figure 8.** Time-dependent photocatalytic gas production profile of the wood under light illumination.

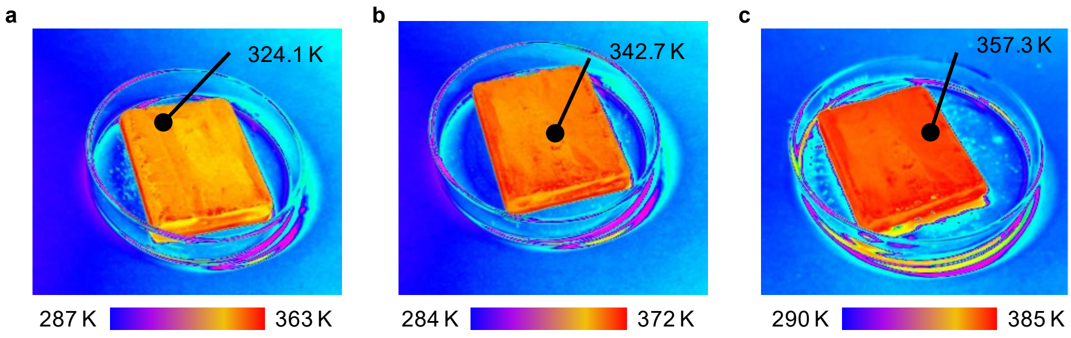


**Supplementary Figure 9.** Infrared radiation thermal images of the wood/CoO system under light illumination with different solar intensity. **a** 100 mW $cm^{-2}$, **b** 200 mW $cm^{-2}$, **c** 300 mW $cm^{-2}$.

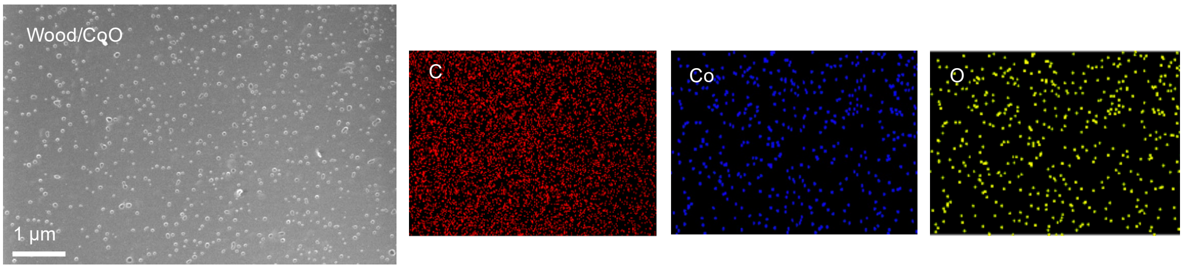


**Supplementary Figure 10.** SEM images and EDS element mapping of CoO NPs attached to the walls of the wood microchannels before photocatalytic reaction.

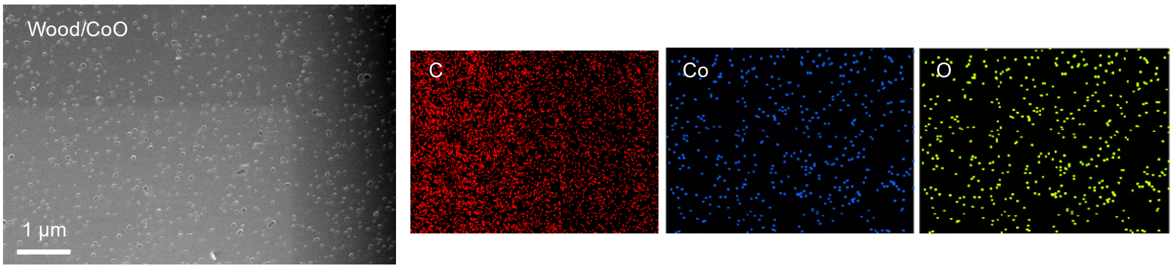


**Supplementary Figure 11.** SEM images and EDS element mapping of CoO attached to the walls of the wood microchannels after the photocatalytic reaction.

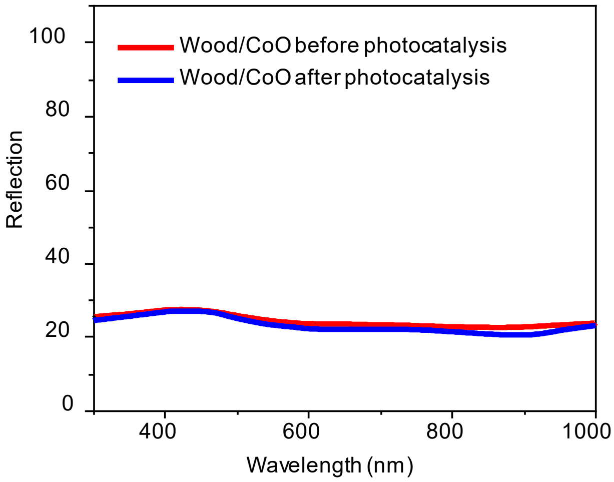


**Supplementary Figure 12.** The reflection spectra wood/CoO system before and after photocatalysis process.

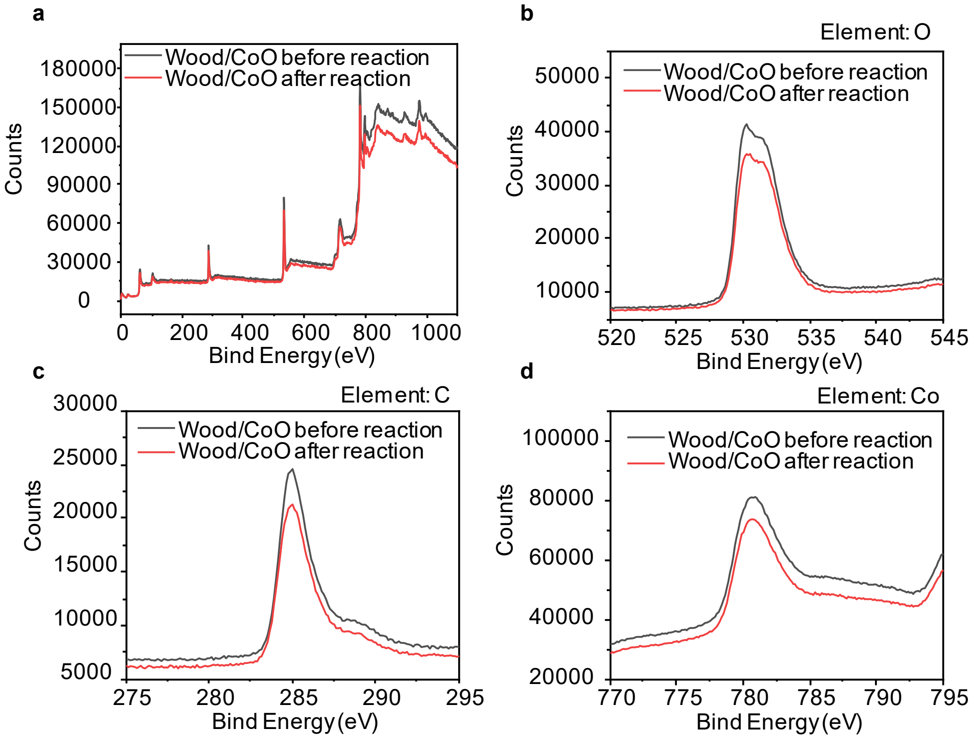


**Supplementary Figure 13.** The XPS spectra wood/CoO system before and after the photocatalysis process. **a** XPS full spectrum of the wood/CoO system before/after the reaction, **b** high-resolution XPS of element O, **c** high-resolution XPS of element C, and **d** high-resolution XPS of element Co for the wood/CoO system.

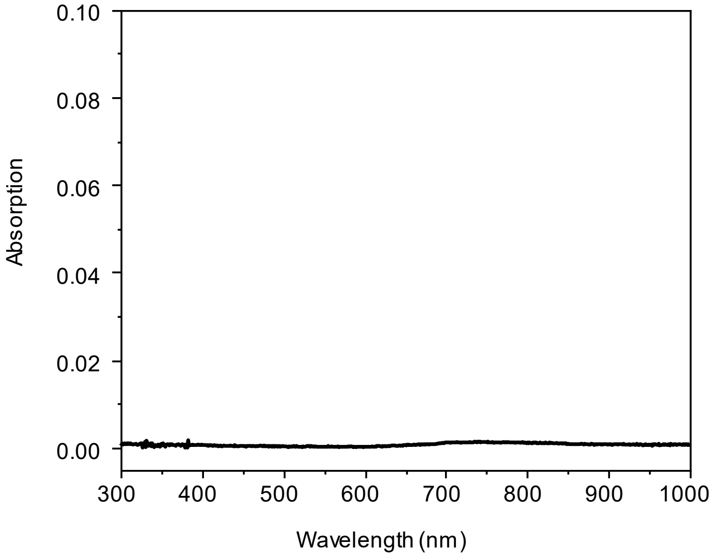


**Supplementary Figure 14**. The absorption spectrum from bulk water in the wood/CoO system after photocatalytic reaction.

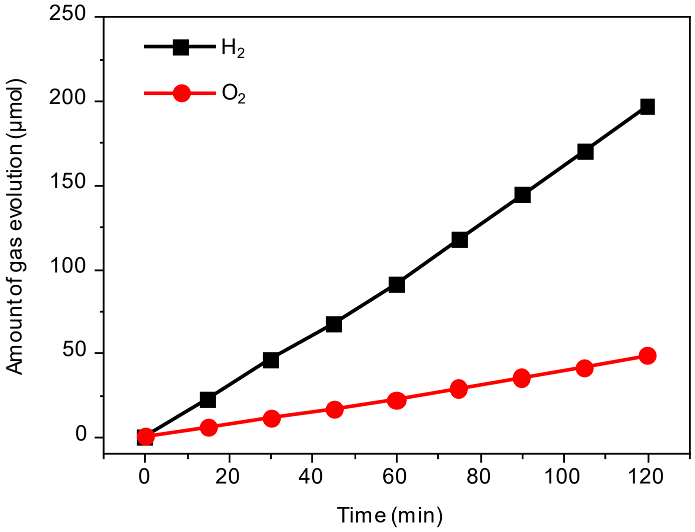


**Supplementary Figure 15.** Time-dependent photocatalytic gas production profiles from the liquid water. The photocatalyst is CoO NPs, and the sacrificial agent is not added.

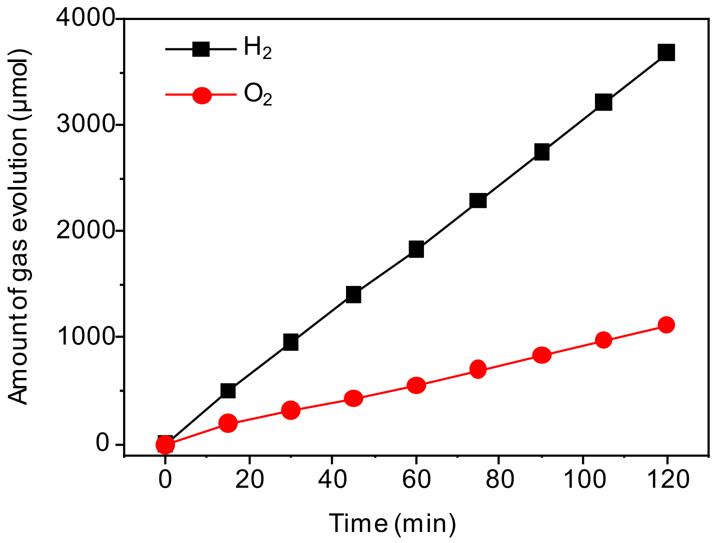


**Supplementary Figure 16.** Time-dependent photocatalytic gas production profiles from the wood/CoO.

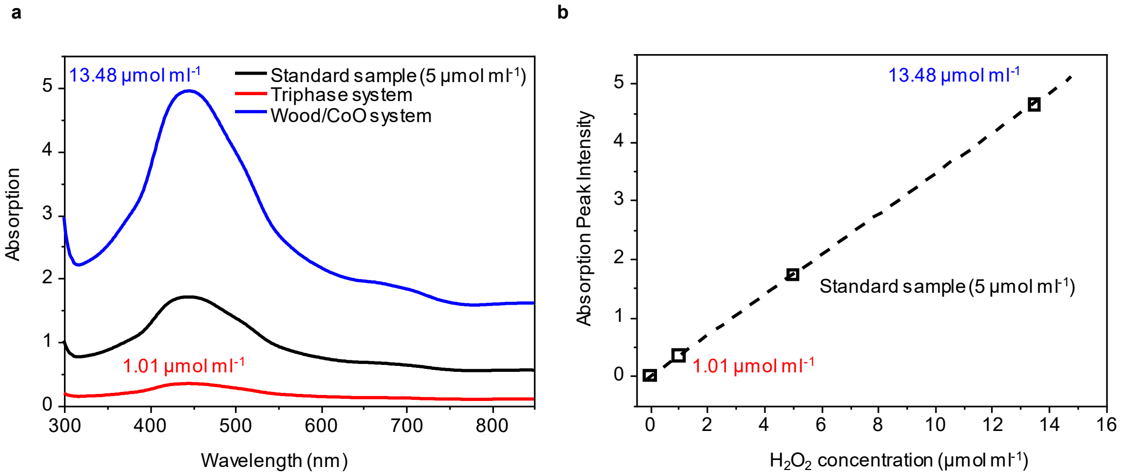


**Supplementary Figure 17.** The $H_2O_2$ concentration determination from absorption spectra. **a** the absorption spectra from different $H_2O_2$ concentration, and **b** the linear fitting between absorption peak and $H_2O_2$ concentration.

The 5 μmol ml$^{-1}$ $H_2O_2$ was used as the standard sample to react with copper (II) sulfate solution and 2,9-dimethyl-1,10-phenanthroline (DMP) solution, and the obtained absorption spectrum was used as the baseline. Due to the linear relationship between absorption peak and $H_2O_2$ concentration [2], the $H_2O_2$ concentration in the solution after the photocatalytic reaction could be calculated based on the above absorption spectra. After the photocatalytic reaction, the concentration of $H_2O_2$ measured is about 1.01 and 13.48 μmol ml$^{-1}$ for the triphase reaction system and wood/CoO systems, respectively.

In the triphase reaction system, after 120 min test, the amount of $H_2$ evolution is about 196.98 μmol, and the amount of $O_2$ evolution is about 48.04 μmol. After the reaction, the $H_2O_2$ concentration is 1.01 μmol ml$^{-1}$ in 100 ml reaction solvent. Thus the amount of $H_2O_2$ is 101 μmol. The $H_2O_2$ converts to the $O_2$ following the reaction equation below:

$$2H_2O_2 \rightarrow 2H_2O + O_2 \quad (1)$$

Combing the amount of directly produced $O_2$ (48.04 μmol) and the amount of indirectly converted $O_2$ from $H_2O_2$ (50.50 μmol), the total amount of $O_2$ is 98.54 μmol. Thus, the production ratio of $H_2$ and $O_2$ is 196.68:98.54 (i.e., 1.99:1).

Regarding the wood/CoO system, the amount of $H_2$ evolution is about 3677.55 μmol, and the amount of $O_2$ evolution is about 1121.21 μmol after 120 min test. The $H_2O_2$ concentration is 13.48 μmol ml$^{-1}$ in 100 ml reaction solvent after the catalytic reaction. Thus, the amount of $H_2O_2$ is 1348 μmol. Combing the amount of directly produced $O_2$ (1121.21 μmol) and the amount of indirectly converted $O_2$ from $H_2O_2$ (674 μmol), the total amount of $O_2$ is 1795.21 μmol. Thus, the converted amount of $O_2$ is 1795.21 μmol. Considering the above calculation, the production ratio of $H_2$ and $O_2$ is 3677.55:1795.21 (i.e., 2.04:1).

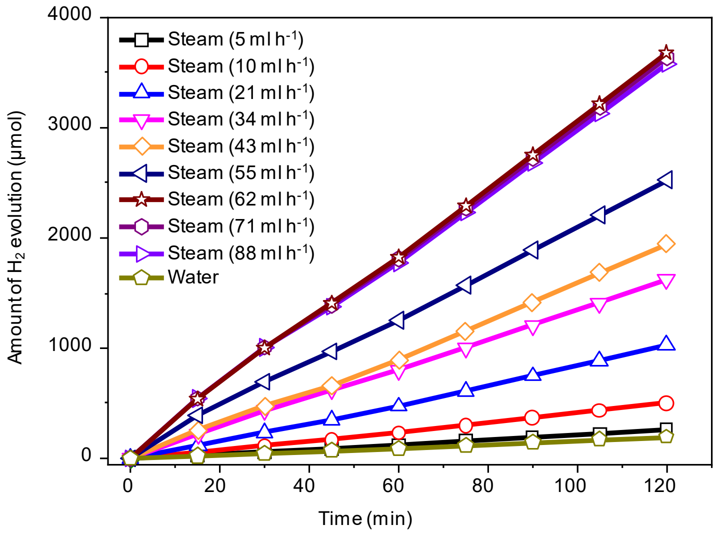


**Supplementary Figure 18.** Time-dependent photocatalytic hydrogen gas production profiles from the liquid water and water steam with different flow rates. The photocatalyst is CoO NPs.

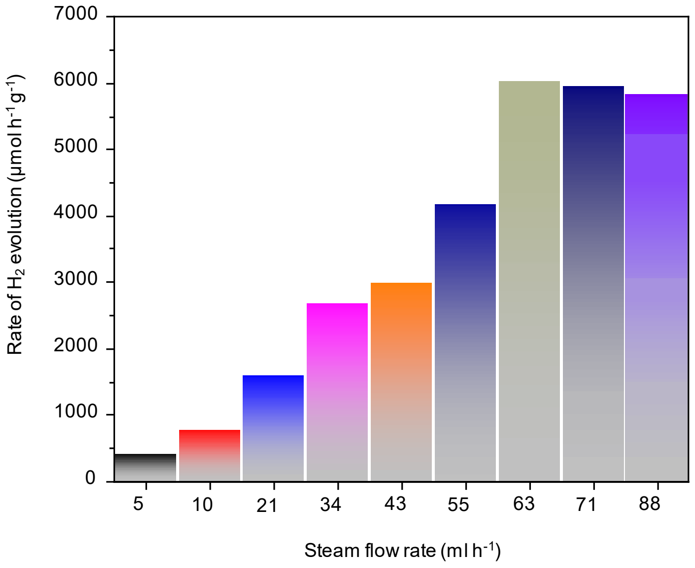


**Supplementary Figure 19.** The hydrogen production rate as a function of flow rate of the water steam. The photocatalyst is CoO NPs.

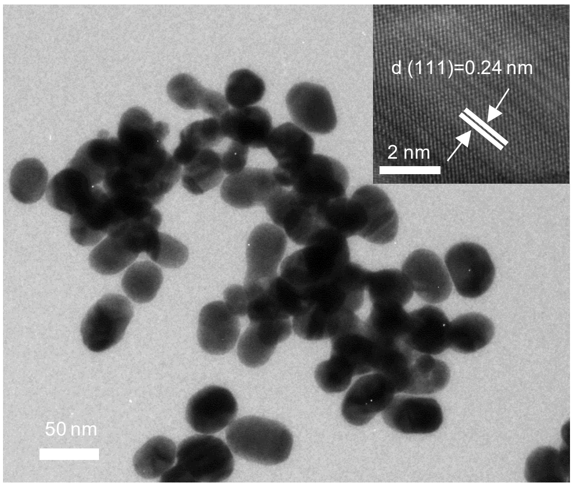


**Supplementary Figure 20.** TEM image of CoO NPs after the photocatalytic reaction. Inset: HRTEM image of a CoO NP.

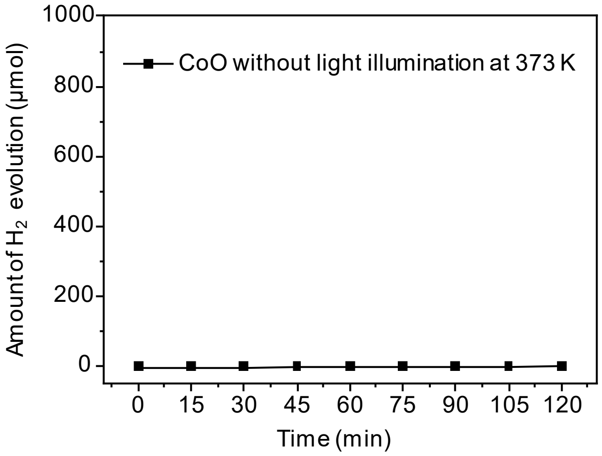


**Supplementary Figure 21.** Time-dependent photocatalytic hydrogen gas production profile from liquid water at the reaction temperature of 373 K without light illumination.

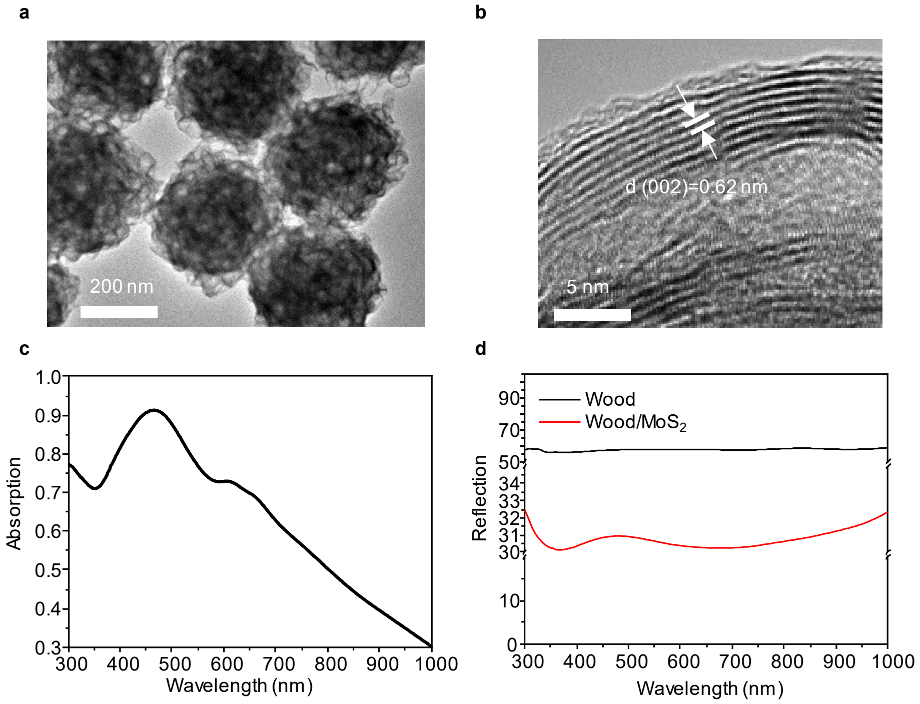


**Supplementary Figure 22. a** TEM image of $MoS_2$. **b** HRTEM image of $MoS_2$. **c** The absorption spectrum of $MoS_2$. **d** Reflection spectra of the wood and wood/$MoS_2$ system.

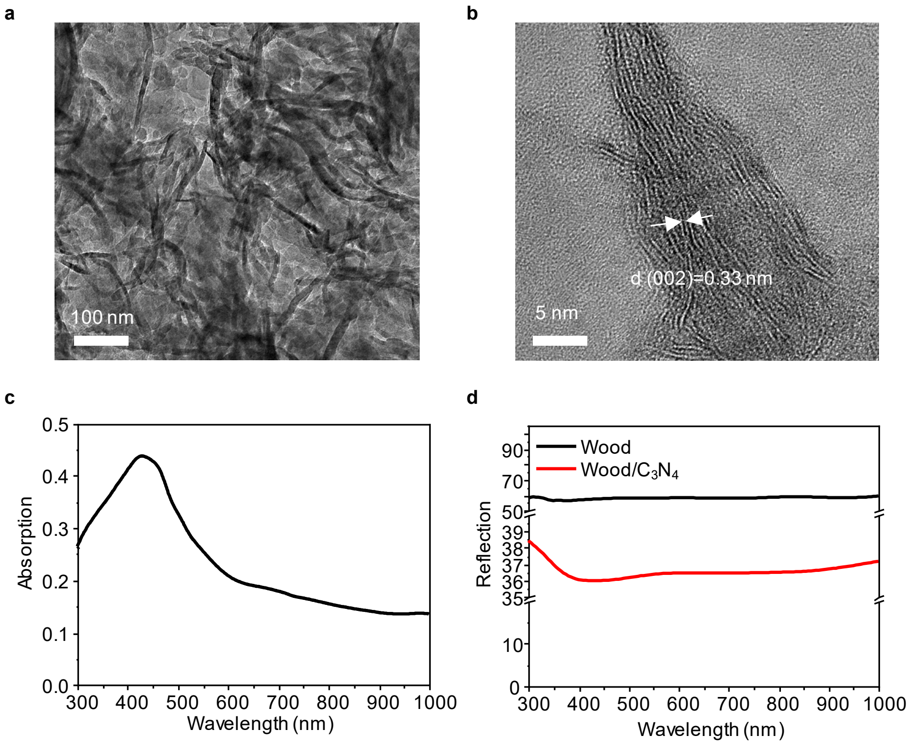


**Supplementary Figure 23. a** TEM image of $C_3N_4$. **b** HRTEM image of $C_3N_4$. **c** The absorption spectrum of $C_3N_4$. **d** Reflection spectra of the wood and wood/$C_3N_4$ system.

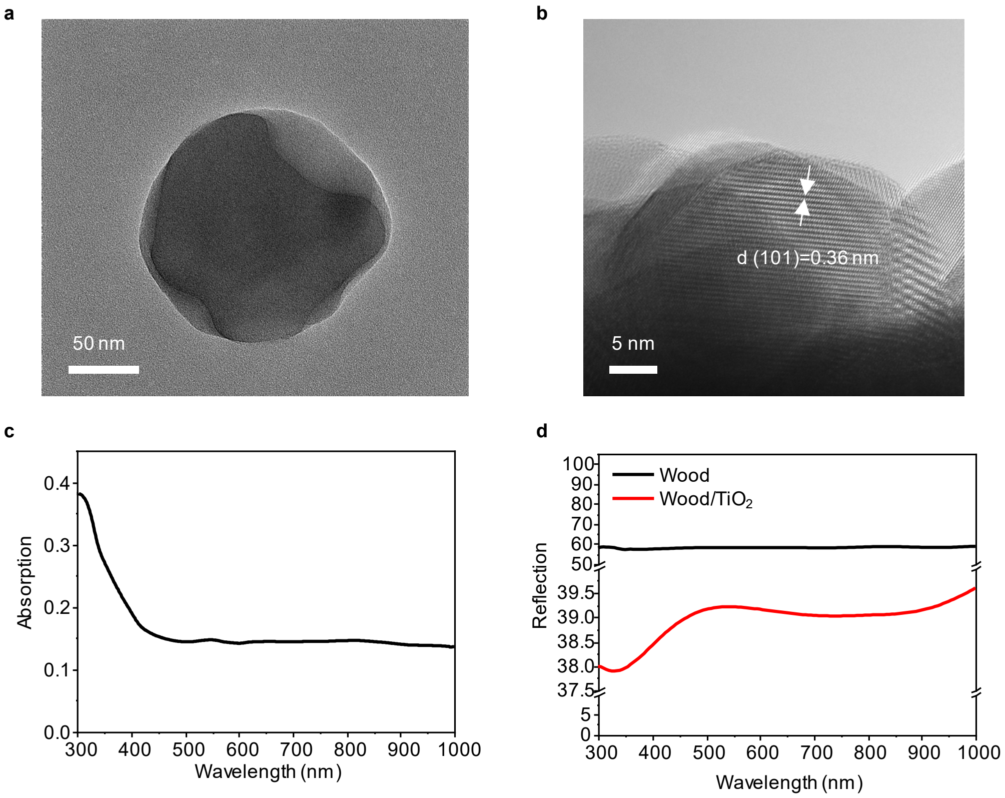


**Supplementary Figure 24. a** TEM image of $TiO_2$. **b** HRTEM image of $TiO_2$. **c** The absorption spectrum of $TiO_2$. **d** Reflection spectra of the wood and wood/$TiO_2$ system.

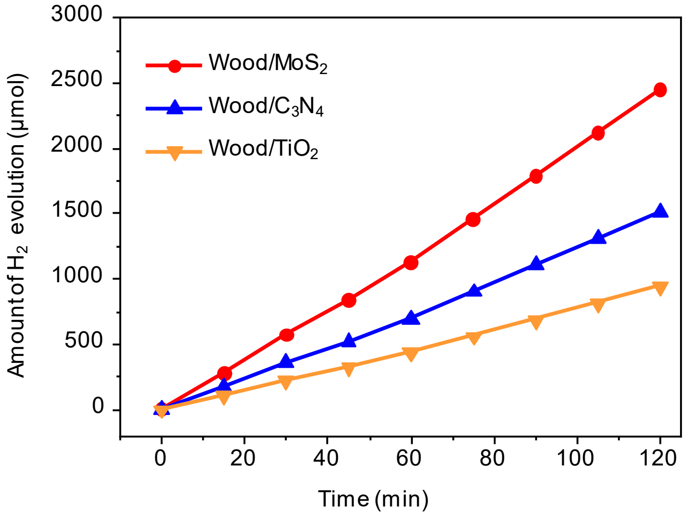


**Supplementary Figure 25.** Amount of hydrogen production for the wood/$MoS_2$, wood/$C_3N_4$, and wood/$TiO_2$ system.

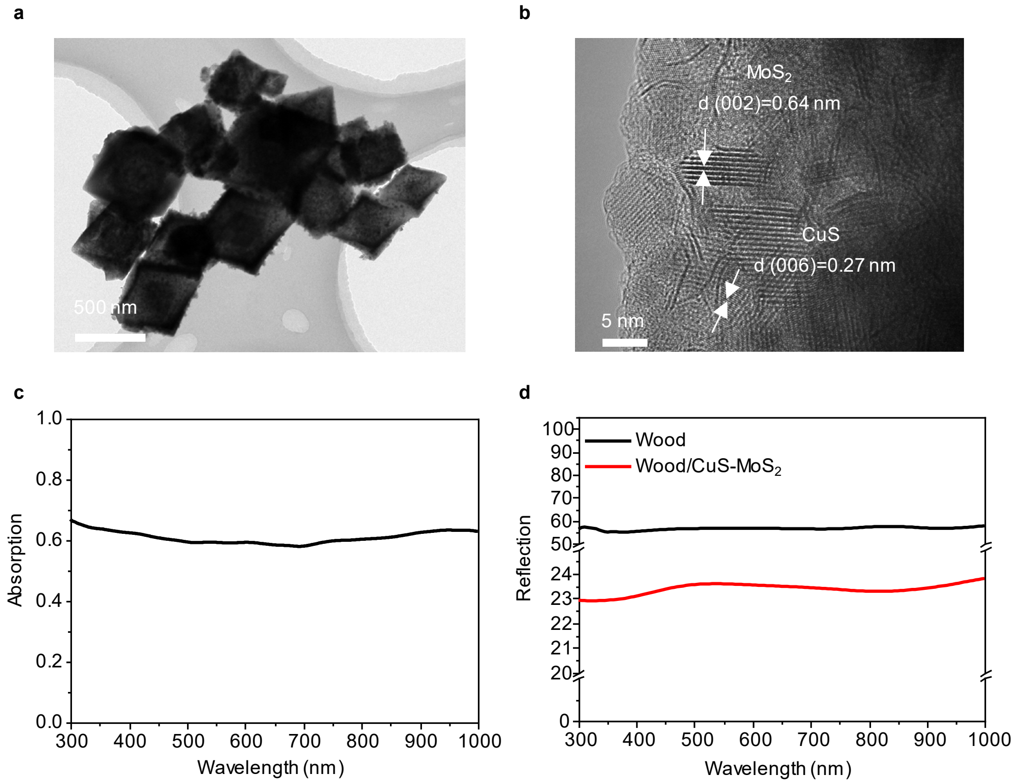


**Supplementary Figure 26. a** TEM image of CuS-$MoS_2$. **b** HRTEM image of CuS-$MoS_2$. **c** The light absorption spectra of CuS-$MoS_2$. **d** Reflection spectra of the wood and wood/CuS-$MoS_2$ system.

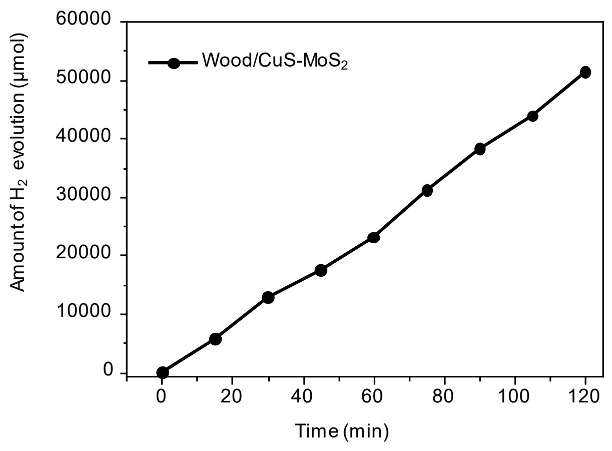


**Supplementary Figure 27.** Amount of hydrogen production for the biphase wood/CuS-$MoS_2$ system without a sacrificial agent.

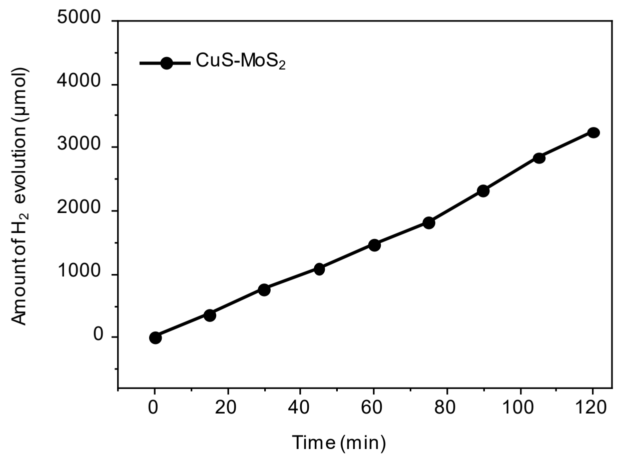


**Supplementary Figure 28.** Amount of hydrogen production for the triphase CuS-$MoS_2$ system without a sacrificial agent.

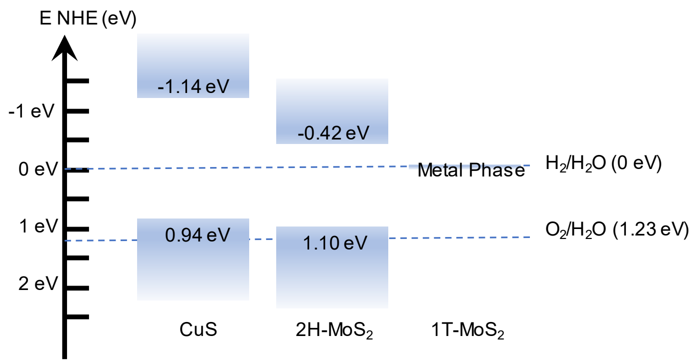


**Supplementary Figure 29.** Scheme of energy band positions of CuS-MoS$_2$ photocatalyst.[3]

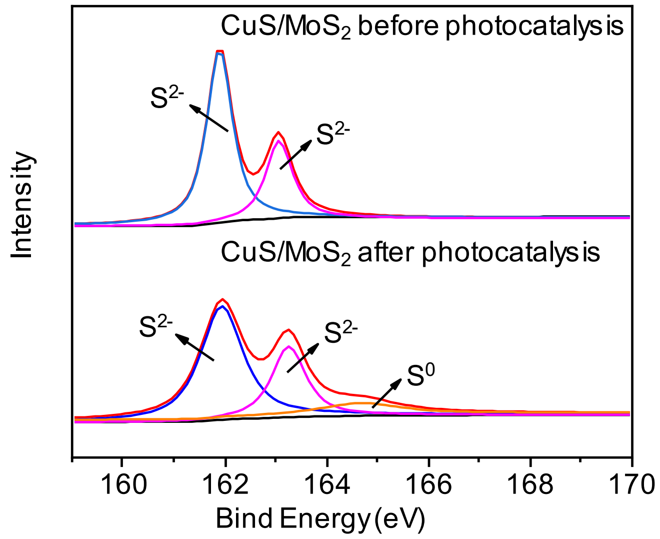


**Supplementary Figure 30.** The high-resolution XPS of element S for CuS/MoS$_2$ before and after photocatalytic reaction.

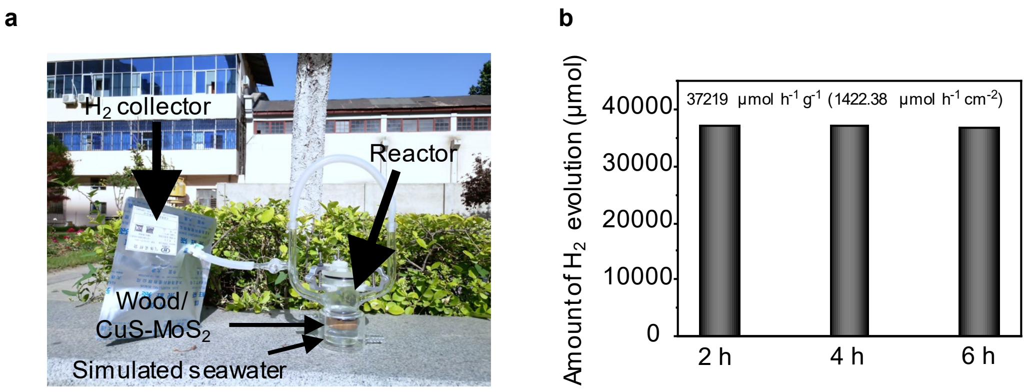


**Supplementary Figure 31.** Hydrogen evolution for the biphase wood/CuS-MoS$_2$ system in simulated seawater. **a** Photograph of hydrogen evolution for the biphase wood/CuS-MoS$_2$ architecture in simulated seawater under the illumination of sunshine. **b** Rate of $H_2$ evolution from the biphase wood/CuS-MoS$_2$ system in simulated seawater. The $H_2$ collector was replaced by a new one every two hours to extract the gas, which was then introduced to the GC device for evaluating the $H_2$ production.

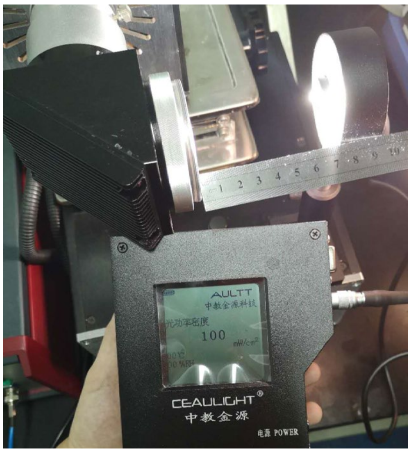


**Supplementary Figure 32**. The photograph of the light intensity measurement.

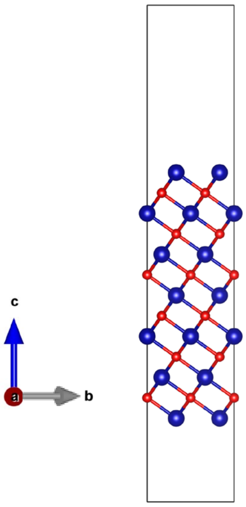


**Supplementary Figure 33.** The model structure schemes of CoO

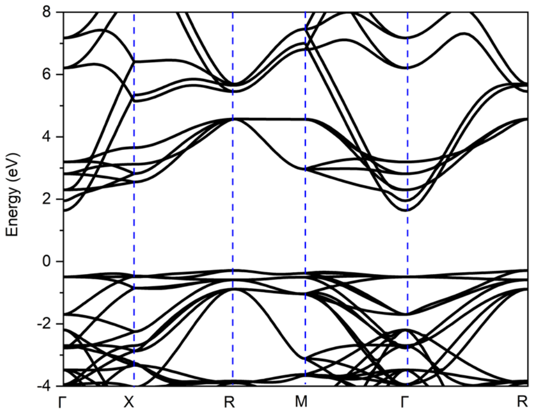


**Supplementary Figure 34.** The band structures of CoO

**Supplementary Table 1.** The element Co concentration in the bulk water from the wood/CoO system after the photocatalytic reaction based on ICP measurement. Three samples are tested.

| | Sample Test 1 | Sample Test 2 | Sample Test 3 |
|---|---|---|---|
| Element Co Concentration (μg $ml^{-1}$) | 0.0102 | 0.0123 | 0.0088 |

**Supplementary Table 2.** The measurement information for AQY calculation, including catalysts, wavelength, light intensity, and $H_2$ yield.

| Catalysts | Wavelength (nm)/ Light intensity (mW $cm^{-2}$) | $H_2$ yield (μmol)/ AQY (%) |
|---|---|---|
| Wood/CoO | 380/13.82 | 255.01/41.1 |
| Wood/CoO | 420/16.32 | 405.85/50.1 |
| Wood/CoO | 500/12.32 | 325.77/44.8 |
| Wood/$MoS_2$ | 380/13.77 | 195.87/31.7 |
| Wood/$MoS_2$ | 420/16.59 | 295.25/35.9 |
| Wood/$MoS_2$ | 500/11.97 | 223.16/31.5 |
| Wood/$C_3N_4$ | 380/13.63 | 122.67/20.0 |
| Wood/$C_3N_4$ | 420/16.12 | 273.49/34.2 |
| Wood/$C_3N_4$ | 500/12.43 | 175.35/23.9 |
| Wood/$TiO_2$ | 360/13.54 | 193.74/33.6 |
| Wood/$TiO_2$ | 380/16.87 | 90.24/11.9 |

## References


1 Xue, G. et al. Robust and low-cost flame-treated wood for high-performance solar steam generation. *ACS Appl. Mater. Inter.* **9**, 15052-15057 (2017).

2 Baga, A. N., Johnson, G. R. A., Nazhat, N. B. & Saadalla-nazhat, R. A. A simple spectrophotometric determination of hydrogen peroxide at low concentrations in aqueous solution. *Anal. Chim. Acta* **204**, 349-353 (1988).

3 Xin, X. et al. In-situ growth of high-content 1T phase $MoS_2$ confined in the CuS nanoframe for efficient photocatalytic hydrogen evolution. *Appl. Catal. B-Environ.* **269**, 118773 (2020).
